# The effects of labor on airway outcomes with Supreme™ laryngeal mask in women undergoing cesarean delivery under general anesthesia: a cohort study

**DOI:** 10.1186/s12871-020-01132-5

**Published:** 2020-08-26

**Authors:** Ming Jian Lim, Hon Sen Tan, Chin Wen Tan, Shi Yang Li, Wei Yu Yao, Yong Jing Yuan, Rehena Sultana, Ban Leong Sng

**Affiliations:** 1 Department of Women’s Anesthesia, KK Women’s and Children’s Hospital, 100 Bukit Timah Road, Singapore, 229899 Singapore; 2 Duke-NUS Medical School, 8 College Road, Singapore, 169857 Singapore; 3 Department of Anesthesiology and Perioperative Medicine, Quanzhou Macare Women’s Hospital, Quanzhou, Fujian Province China; 4 Department of Anesthesiology, Qinghai University Affiliated Hospital, Xining, Qinghai Province China; 5 Centre for Quantitative Medicine, Duke-NUS Medical School, 8 College Road, Singapore, 169857 Singapore

**Keywords:** Obstetrics, Mallampati score, Airway

## Abstract

**Background:**

Pregnancy is associated with higher incidence of failed endotracheal intubation and is exacerbated by labor. However, the influence of labor on airway outcomes with laryngeal mask airway (LMA) for cesarean delivery is unknown.

**Methods:**

This is a secondary analysis of a prospective cohort study on LMA use during cesarean delivery. Healthy parturients who fasted > 4 h undergoing Category 2 or 3 cesarean delivery with Supreme™ LMA (sLMA) under general anesthesia were included. We excluded parturients with BMI > 35 kg/m^2^, gastroesophageal reflux disease, or potentially difficult airway (Mallampati score of 4, upper respiratory tract or neck pathology). Anesthesia and airway management reflected clinical standard at the study center. After rapid sequence induction and cricoid pressure, sLMA was inserted as per manufacturer’s recommendations. Our primary outcome was time to effective ventilation (time from when sLMA was picked up until appearance of end-tidal carbon dioxide capnography), and secondary outcomes include first-attempt insertion failure, oxygen saturation, ventilation parameters, mucosal trauma, pulmonary aspiration, and Apgar scores. Differences between labor status were tested using Student’s t-test, Mann-Whitney U test, or Fisher’s exact test, as appropriate. Quantitative associations between labor status and outcomes were determined using univariate logistic regression analysis.

**Results:**

Data from 584 parturients were analyzed, with 37.8% in labor. Labor did not significantly affect time to effective ventilation (mean (SD) for labor: 16.0 (5.75) seconds; no labor: 15.3 (3.35); mean difference: -0.65 (95%CI: − 1.49 to 0.18); *p* = 0.1262). However, labor was associated with increased first-attempt insertion failure and blood on sLMA surface. No reduction in oxygen saturation or pulmonary aspiration was noted.

**Conclusions:**

Although no significant increase in time to effective ventilation was noted, labor may increase the number of insertion attempts and oropharyngeal trauma with sLMA use for cesarean delivery in parturients at low risk of difficult airway. Future studies should investigate the effects of labor on LMA use in high risk parturients.

**Trial registration:**

The study was prospectively registered at clinicaltrials.gov (NCT02026882) on 3 January 2014.

## Background

Pregnancy is associated with higher risk of failed endotracheal intubation, with an estimated incidence of 1:250 compared to 1:2000 in non-pregnant patients [1, 2]. Although recent reports from the Mothers and Babies: Reducing Risk through Audits and Confidential Enquiries (MBRRACE-UK) have shown a reduction in anesthesia-related deaths [3], hypoxia resulting from failure to intubate or ventilate is a consistent cause of maternal mortality. Airway-related mortality occurs in 2.3 per 100,000 cesarean deliveries under general anesthesia compared to 1 per 180,000 in the general surgical population [4], which may be exacerbated by declining use of general anesthesia for cesarean delivery and concomitant reduction in training and experience with endotracheal intubation in obstetrics [4, 5].

Labor has been associated with anatomical changes that increase the likelihood of difficult intubation, and Mallampati scores after labor were 1 to 2 grades higher compared to pre-labor, with a greater proportion of parturients possessing Mallampati scores of 3 or 4 [6, 7]. The Mallampati score is a common bedside airway assessment used to predict difficult intubation [8]; with scores of 3 or 4 corresponding to relative risks of 7.6 and 11.3 for difficult intubation compared to a score of 1, respectively [9]. Moreover, labor significantly decreases oropharyngeal area and volume, which may further impede endotracheal intubation [6]. These anatomical changes are attributed to laryngeal edema arising from rapid intravenous fluid administration, antidiuretic effects of oxytocin, and prolonged straining during labor [10].

Despite concerns that labor may increase the risk of difficult endotracheal intubation, to our knowledge the effects of labor on laryngeal mask airway (LMA) use during cesarean delivery have not been elucidated. This is of particular importance given the recent recommendations of the LMA as a second-line or “rescue” airway device in the event of failed endotracheal intubation [1, 2, 11–13]. In fact, obstetric airway management guidelines have specifically recommended the use of second-generation LMAs to maintain ventilation and oxygenation in the event of failed endotracheal intubation [14]. Second-generation LMAs such as Supreme™ contain a separate channel to isolate the gastrointestinal tract with high sealing pressures and reduce the risk of pulmonary aspiration if they are well positioned [15–17]. Subsequent studies have demonstrated the efficacy and safety of the Supreme™ LMA (sLMA) as an alternative to endotracheal intubation for selected parturients undergoing cesarean delivery [18–20]. However, notwithstanding the utility of the LMA as a rescue airway device, LMA use in pregnant parturients is associated with a first-attempt failure rate of 2% [18, 19], and underscores the importance of identifying perinatal factors that may increase the likelihood of LMA failure. Therefore, the objective of this study is to investigate the potential effects of labor on airway outcomes with the use of sLMA for cesarean delivery under general anesthesia. Our primary outcome is time to effective ventilation, and secondary outcomes include oxygenation and ventilation parameters, seal pressure, and oropharyngeal mucosal trauma.

## Methods

This is a secondary analysis of a prospective cohort study investigating the use of sLMA during cesarean delivery [18]. With this dataset, we had previously published the association of Mallampati scores on airway outcomes with sLMA use for cesarean delivery [21]. Approval was obtained from the Institutional Review Board at the Quanzhou Women’s and Children’s Hospital, Fujian Province, China, (dated 11 Nov 2013) and registered with clinicaltrials.gov (NCT02026882) on 3 January 2014.

Analysis was performed on data from 584 parturients, enrolled between January 2014 to December 2014 at Quanzhou Women’s and Children’s Hospital. At this center, approximately 35% of parturients undergo cesarean delivery mostly due to maternal request, with the majority of cases performed under general anesthesia using the sLMA as the airway device of choice. Enrolled parturients were American Society of Anesthesiologists (ASA) physical status classification I to III, underwent Category 2 or 3 cesarean delivery under general anesthesia, and had fasted for 4 or more hours. We excluded parturients with BMI > 35 kg/m^2^, underwent cesarean delivery under regional anesthesia, had known gastroesophageal reflux disease, or with potentially difficult airway defined as having Mallampati score of 4, upper respiratory tract or neck pathology. The parturients were analyzed according to the presence or absence of labor before cesarean delivery, defined as the presence of painful uterine contractions associated with cervical dilation [22].

Anesthesia and airway management reflects the clinical standard at the study center. All parturients were given intravenous ranitidine for aspiration prophylaxis, and electrocardiogram, pulse oximetry, capnography, and non-invasive blood pressure monitors were applied. After preoxygenation for 3 min, a rapid sequence induction with intravenous propofol (2–3 mg/kg), succinylcholine (100 mg) and application of cricoid pressure by a trained anesthetic assistant was performed, followed by sLMA insertion. All sLMA were inserted using the recommended single-handed rotational technique, and were performed by three investigators (Yao, Li, and Yuan), each with more than 5 years of experience in sLMA use for cesarean delivery. sLMA size was chosen according to manufacturer’s guidelines but can be changed to a more appropriate size according to the discretion of the anesthesiologist. Cricoid pressure was released upon inflation of the sLMA cuff with a manometer to 60 cmH_2_O and confirmation of the ability to ventilate via auscultation of breath sounds and presence of end-tidal carbon dioxide with capnography. Airway maneuvers to assist sLMA insertion such as head-tilt or jaw thrust were permitted. The time to effective ventilation, defined as the time from when the sLMA was picked up until the appearance of end-tidal carbon dioxide capnography, and number of attempts at sLMA insertion with each attempt defined as complete insertion and removal of the sLMA, were recorded. Next, a pre-mounted #14 orogastric tube was advanced through the gastric drainage port of the sLMA. After confirmation of adequate placement by aspiration of gastric contents and auscultation of a “swoosh” over the epigastric area with injection of 5 mL of air, suctioning of the orogastric tube was performed. Lastly, sLMA seal pressure was measured by closing the adjustable pressure limiting valve while maintaining 3 L/min fresh gas flow in a closed circle circuit and observing the airway pressure at equilibrium.

Cesarean delivery was allowed to commence if the following criteria were met: presence of a square-wave capnograph, sLMA cuff pressure of 60 cmH_2_O, sLMA bite block position located between the incisors, adequately-positioned orogastric tube, and seal pressure of > 20 cmH_2_O. Endotracheal intubation would be performed if sLMA insertion was not successful after two attempts, took more than 1 min, or desaturation occurred (oxygen saturation < 92%). All parturients were positioned in left lateral tilt using a wedge. Rocuronium (0.5 mg/kg) was given to maintain muscle relaxation, and anesthesia was maintained with 1.5 to 2% sevoflurane and 50% mix of nitrous oxide in oxygen. Mechanical ventilation was instituted with a tidal volume of 6 to 10 ml/kg and respiratory rate of 10 to 16 breaths/min. The incidence of airway complications, defined as airway obstruction, inadequate oxygenation or ventilation, bronchospasm, laryngospasm and clinical signs of pulmonary aspiration including hypoxemia, auscultation of wheezing or crepitations, and postoperative dyspnea were recorded. The obstetricians were advised to avoid excessive fundal pressure during delivery of the fetus. Upon completion of surgery, the orogastric tube was suctioned and removed, and the sLMA was withdrawn and inspected for the presence of blood. An independent assessor reviewed the patient before discharge from the post-anesthesia care unit to record the incidence of sore throat and voice hoarseness.

The primary airway outcome is time to effective ventilation and secondary outcomes include first-attempt sLMA insertion failure, oropharyngeal leak pressure, peak airway pressure, lowest oxygen saturation during sLMA insertion, volume and pH of gastric aspirate, pH of the sLMA laryngeal surface.

### Statistical analysis

All demographic, anesthetic, and clinical were summarized based on parturient’s labor status. Categorical data were summarized as frequency with the corresponding proportion, while continuous variables were presented as mean (standard deviation (SD)) or median (interquartile range (IQR)), as appropriate. Differences between labor status for continuous data were tested using Student’s t-test or Mann-Whitney U test, whichever appropriate, while categorical data was tested using the Fisher’s exact test. Univariate logistic regression analysis was used to express quantitative association between labor status and other factors. Associations from logistic regression analysis were expressed as odds ratios (OR) with 95% confidence intervals (95%CI). Time to effective ventilation (primary outcome), oropharyngeal leak pressure, peak airway pressure, lowest oxygen saturation during sLMA insertion, volume and pH of gastric aspirate, and pH of the sLMA laryngeal surface were treated as continuous data. First-attempt sLMA insertion failure was treated as binary data. Significance level was set at *p* < 0.05 and all tests were two-sided. SAS 9.4 software (SAS Institute Inc., Cary, NC, USA) was used for all analysis. A post-hoc power calculation showed that we had 95% power to detect a difference of 2 s in time to effective ventilation with SD of 5, allocation ratio as 1:1, an alpha error of 0.05 and two-sided significance.

## Results

Data from all 584 parturients enrolled in the prospective cohort study were analyzed, of whom 221 (37.8%) were in labor and 363 (62.2%) were not in labor. There was no withdrawal or dropout. Parturient, obstetric, fetal, and surgical characteristics are summarized in Table 1. Labor was associated with significantly lower maternal weight, gestational age, and fetal weight. In addition, labor was associated with increased Category 2 cesarean delivery and longer surgical duration. Of note, there was no significant association between labor status and Mallampati scores.

**Table 1:** Parturient, fetal and surgical characteristics, and univariate associations with labor status

Characteristics	Labor Status		Univariate analysis	
	Labor ***N*** = 221	No Labor ***N*** = 363	Unadjusted OR (95% CI)	P - value
Age (years), mean (SD)	28.5 (4.19)	29.1 (4.10)	0.97 (0.93 to 1.01)	0.1136
Weight (kg), mean (SD)	66.7 (7.84)	69.8 (9.88)	0.96 (0.95 to 0.99)	0.0001
Height (m), mean (SD)	1.6 (0.12)	1.6 (0.07)	0.71 (0.11 to 4.48)	0.7109
ASA status, mean (SD)	1.8 (0.44)	1.8 (0.44)	1.04 (0.71 to 1.52)	0.8571
Mallampati score, mean (SD)	1.7 (0.67)	1.8 (0.66)	0.86 (0.66 to 1.10)	0.2257
Baseline SBP (mmHg), mean (SD)	123.2 (14.44)	122.1 (11.88)	1.01 (0.99 to 1.02)	0.3151
Duration of surgery (min), mean (SD)	30.8 (9.96)	28.7 (9.01)	1.02 (1.01 to 1.04)	0.0101
Cesarean, n (%)				< 0.0001
Category 2	169 (76.5)	24 (6.6)	Reference	–
Category 3	52 (23.5)	339 (93.4)	45.91 (27.36 to 77.04)	–
Gestation (weeks), mean (SD)	37.1 (2.52)	38.4 (1.15)	0.67 (0.60 to 0.75)	< 0.0001
Fetal weight (g), mean (SD)	2766 (578)	3167 (447)	0.998 (0.998 to 0.999)	< 0.0001

Abbreviations: *ASA* American Society of Anesthesiologists, *SBP* Systolic blood pressure

Airway outcomes with sLMA insertion were summarized in Table 2. Laboring parturients had mean time to effective ventilation of 16.0 (SD 5.75) seconds with sLMA insertion, compared to 15.3 (SD 3.35) seconds in non-laboring parturients. Based on univariate analysis, presence of labor was not associated with significant change in our primary outcome of time to effective ventilation, with a mean reduction of 0.65 s (95%CI − 1.49 to 0.18, *p* = 0.1262).

**Table 2:** Airway outcomes with sLMA insertion and univariate associations with labor status

Continuous variables	Labor Status		Univariate analysis	
	Labor ***N*** = 221	No Labor ***N*** = 363	Mean difference (95% CI)	***p***-value
Time to effective ventilation (s), mean (SD)	16.0 (5.75)	15.3 (3.35)	-0.65 (−1.49 to 0.18)	0.1262
Seal pressure (cmH_2_O), mean (SD)	26.8 (3.44)	27.5 (3.87)	0.79 (0.19 to 1.4)	0.0104
Peak airway pressure (cmH_2_O), mean (SD)	17.3 (3.76)	18.9 (4.03)	1.54 (0.89 to 2.19)	< 0.0001
Minimum tidal volume (ml), mean (SD)	465.0 (45.62)	484.2 (57.12)	19.17 (10.75 to 27.6)	< 0.0001
Maximum tidal volume (ml), mean (SD)	477.9 (42.61)	501.1 (52.63)	23.21 (15.39 to 31.03)	< 0.0001
Lowest SpO_2_ (%), mean (SD)	98.6 (1.10)	98.5 (1.14)	−0.10 (−0.29 to 0.09)	0.3012
Gastric aspirate volume (mL), mean (SD)	12.0 (7.16)	15.5 (17.12)	3.53 (1.53 to 5.54)	0.0006
pH of gastric aspirate, mean (SD)	2.3 (0.62)	2.4 (0.95)	0.11 (−0.02 to 0.24)	0.0851
pH of sLMA laryngeal surface, mean (SD)	7.0 (0.55)	7.1 (0.39)	0.08 (0.00 to 0.16)	0.0559
Binary variables	**Labor** ***N*** **= 221**	**No Labor** ***N*** **= 363**	**Unadjusted odds ratio** **(95%CI)**	***p*-value**
First-attempt sLMA insertion failure, n (%)	9 (4.07)	1 (0.28)	15.37 (1.93 to 122.14)	0.0098
Blood on sLMA, n (%)	7 (3.17)	1 (0.28)	11.84 (1.45 to 96.82)	0.0212
Sore throat, n (%)	14 (6.33)	24 (6.61)	0.96 (0.48 to 1.89)	0.8959
Voice hoarseness, n (%)	2 (0.90)	2 (0.55)	1.65 (0.23 to 11.79)	0.6185

However, labor was associated with increased first-attempt sLMA insertion failure, although all sLMA insertions were successful with a maximum of two attempts. In addition, laboring parturients were found to have significantly lower seal and peak airway pressures, decreased minimum and maximum tidal volumes, lower gastric aspirate volume, lower sLMA laryngeal surface pH, and increased incidence of blood on sLMA. There was no significant change in lowest oxygen saturation and incidence of sore throat or voice hoarseness. No episodes of bronchospasm, laryngospasm, or pulmonary aspiration were noted in either group.

Maternal and fetal outcomes are summarized in Table 3. Presence of labor was associated with lower 1- and 5-min Apgar scores, and reduced patient satisfaction. No significant change in umbilical venous cord pH was noted.

**Table 3:** Maternal and fetal outcomes, and univariate associations with labor status

Fetal outcomes	Labor Status		Univariate analysis	
	Labor ***N*** = 221	No Labor ***N*** = 363	Unadjusted odds ratio (95% CI)	***p***-value
Venous cord pH, mean (SD)	7.3 (0.05)	7.3 (0.06)	0.08 (0.00 to 1.80)	0.1110
1-min fetal Apgar, mean (SD)	8.7 (1.47)	9.4 (0.76)	0.52 (0.43 to 0.63)	< 0.0001
5-min fetal Apgar, mean (SD)	9.4 (1.05)	9.9 (0.30)	0.24 (0.16 to 0.34)	< 0.0001
Patient satisfaction (0–100%), mean (SD)	84.3 (9.75)	87.2 (7.64)	0.96 (0.48 to 1.89)	0.0001

## Discussion

In our study cohort of 584 parturients, 37.8% were in labor while 62.2% were not in labor. Labor was not associated with a significant difference in time to effective ventilation. However, labor was associated with significantly increased incidence of first-attempt sLMA insertion failure, lower seal pressure, lower peak airway pressure, and decreased maximum and minimum tidal volumes, albeit without significant reduction in oxygen saturation. No episodes of pulmonary aspiration was noted. Labor also increased the incidence of blood on the sLMA, but without corresponding change in sore throat or voice hoarseness. In addition, 1- and 5-min Apgar scores were reduced, but with no significant change in umbilical venous cord pH.

To our knowledge, this is the first study that investigated the effects of labor on airway outcomes during sLMA use for cesarean delivery. We noted that laboring parturients had significantly higher first-attempt sLMA insertion failure (4.1%) compared to non-laboring parturients (0.3%), but without concomitant increase in time to effective ventilation or desaturation. Nonetheless, the first-attempt insertion failure rate in laboring parturients was double the incidence of 2% reported by other studies that did not account for labor status [19, 23]. Higher first attempt insertion failure rate will likely increase the time to establishment of anesthesia for cesarean delivery which was not accounted for in other studies [24, 25]. Furthermore, successful sLMA insertion was achieved after a maximum of two attempts in our study population, but we should be cognizant that high risk parturients with Mallampati score of 4, upper respiratory tract or neck pathology were excluded from our study. Hence, the effects of labor on time to effective ventilation and first-attempt insertion failure in high-risk difficult obstetric airway should be investigated in future studies.

Labor was associated with significant reduction in seal pressure, peak airway pressure, and minimum and maximum tidal volumes. However, the reduction in tidal volumes are unlikely to be due to the reduction in sLMA seal pressure, given the clinically insignificant mean difference of 0.8 cmH_2_O, and that peak airway pressures did not exceed seal pressures in either group. Instead, the observed difference in tidal volumes may be due to the lower maternal weight in the laboring group, since tidal volumes could be adjusted according to body weight.

We did not find a significant change in Mallampati scores in laboring parturients, in contrast to other studies where Mallampati scores were found to increase 1 to 2 grades in laboring parturients [6, 7]. However, Boutonnet et al. reported that Mallampati scores remain unchanged for 37% of parturients in labor [7], and our study may not be adequately powered to detect a significant change in Mallampati scores. Nonetheless, we have previously shown that Mallampati scores of 3 or 4 did not significantly affect time to effective ventilation, first attempt failure rate, or sLMA seal pressure compared to parturients with Mallampati scores of 1 or 2 undergoing cesarean delivery [21].

The higher incidence of blood on the sLMA suggests that labor increases the risk of oropharyngeal trauma during sLMA insertion, but without corresponding increase in the incidence of sore throat or voice hoarseness. The increase in oropharyngeal trauma may be attributed to fluid accumulation and increased airway edema that occur during labor [6, 26] and possibly associated with the increased number of sLMA insertion attempts in laboring parturients.

Interestingly, gastric aspirate volume was significantly reduced in laboring parturients. This difference may reflect a change in gastric emptying time. Traditionally, pregnancy and labor has been hypothesized to impair gastric motility and emptying, but this has been challenged recently [27], with guidelines even encouraging fluid intake during labor [28]. In early labor, the rate of gastric emptying has been shown to remain unchanged or increase, while advanced labor is associated with delayed gastric emptying [29]. Information on cervical dilation was not collected in this study, and hence we are unable to comment on the stage of labor at the time of cesarean delivery. Nonetheless, the use of LMA in pregnancy raises concern of exacerbating the risk of gastric regurgitation and pulmonary aspiration. Although this study was not powered to investigate the risk of pulmonary aspiration, no episodes of clinical aspiration were detected. Furthermore, the sLMA surface pH, being a surrogate indicator of possible gastric regurgitation, did not reflect that of gastric content.

The use of sLMA in laboring parturients was associated with reduced 1- and 5-min Apgar scores. Of note, the lack of significant reduction in maternal oxygen saturation during sLMA insertion suggests that maternal hypoxemia is unlikely to be the cause of reduced Apgar scores. Instead, the reduction in Apgar scores may be related to the clinical indication prompting urgent cesarean delivery, as demonstrated by the higher proportion of Category 2 cesarean deliveries in laboring parturients. Nonetheless, labor was not associated with significant change in umbilical venous pH, which is arguably a more objective assessment of fetal status, due to the subjectivity of the Apgar score [30].

We acknowledge several limitations with our study. The cesarean delivery rate at the study center is 35%, and sLMA is used for over 2000 deliveries annually. Hence, familiarity with the use of sLMA could have influenced the time to effective ventilation and first-attempt insertion success rate, and these findings may not be applicable to other centers. Cricoid pressure was applied by anesthetic assistants according to routine hospital practice, who were trained to be consistent in this technique, however, the amount of cricoid pressure was not directly measured. In addition, there was no reliable method of blinding the anesthesiologists and the healthcare team on the labor status of the study parturients, which may have influenced our results. The use of sLMA in parturients undergoing general anesthesia raises concerns of gastric regurgitation and pulmonary aspiration. Although we did not detect any clinical signs of pulmonary aspiration or regurgitation, this study was not powered to detect these outcomes. Finally, we excluded parturients with high risk of difficult airway, hence our results may not apply to these parturients.

## Conclusions

In summary, our study found that labor is not associated with significant change in time to effective ventilation when sLMA was used in general anesthesia for cesarean delivery. However, laboring parturients had increased incidence of first-attempt sLMA insertion failure and oropharyngeal trauma, compared to non-laboring parturients. No reduction in oxygen saturation or episodes of pulmonary aspiration were noted. Further research is needed to determine the effects of labor on sLMA use in parturients at higher risk of difficult airway.

## Data Availability

The datasets generated and analyzed during the current study are not publicly available, but are available from the corresponding author on reasonable request.

## Abbreviations

ASA: American Society of Anesthesiologists
CI: Confidence intervals
IQR: Inter-quartile range
MBRRACE-UK: Mothers and Babies: Reducing Risk through Audits and Confidential Enquiries
OR: Odds ratio
LMA: Laryngeal mask airway
sLMA: Supreme™ LMA
SD: Standard deviation
